# 1649. Tracking and reporting antibiotic use: Rising antibiotic prescriptions for URI illness in children during the COVID-19 pandemic.

**DOI:** 10.1093/ofid/ofad500.1483

**Published:** 2023-11-27

**Authors:** Emily Clemons, Matthew Whiteman, Lisa Garavaglia, Shipra Gupta

**Affiliations:** WVU, morgantown, West Virginia; WVU Medicine Children's Hospital, Morgantown, West Virginia; West Virginia University, Morgantown, West Virginia; West Virginia University, Morgantown, West Virginia

## Abstract

**Background:**

During COVID-19 there was an increase in antibiotic utilization across all settings. West Virginia has the highest outpatient antibiotic prescribing rate in the country. As part of our outpatient antibiotic stewardship initiative, we developed and utilized a dashboard to track and report prescribing data in two pediatric clinics in a large health system.

**Methods:**

We reviewed prescribing data from Oct 2020- Mar 2022 in two outpatient pediatric clinics using a dashboard within the electronic medical record. Both clinics are affiliated with a large health system but clinic A was academic and clinic B was non-academic clinic. We also collected data on total number of visits and visits that had antibiotics prescribed. We reviewed prescription data on visits that were diagnosed with upper respiratory tract illness (URI) like acute otitis media, acute sinusitis, acute pharyngitis etc. We compared URI antibiotic prescriptions in Jan 2021-Mar 2021 with Jan 2022- Mar 2022.

**Results:**

There were more than 15,000 visits during the study period. A steady increase in antibiotic prescriptions written per visit was noted in both clinics (Figure 1). There was a significant increase in percentage of prescriptions for URI in the respiratory season for 2021 and 2022 (30.2% vs. 47.7%, p< 0.0001). The most common diagnosis for antibiotic prescription in both clinics was acute otitis media (67 and 72%, respectively). However, clinic B wrote cefdinir prescriptions for 38 % cases of otitis media vs. 12% in clinic A.

**Figure:**
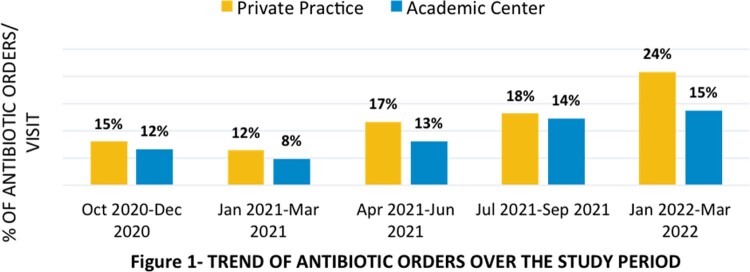

**Conclusion:**

There was an increase in overall prescriptions for antibiotics and prescriptions for URI in both clinic sites. This was most likely due to the prolonged viral season following cessation of COVID-19 mitigation measures. There were significant differences noted in prescribing practices, like cefdinir overuse for treatment of otitis media, which will guide our antimicrobial stewardship interventions in future.

**Disclosures:**

**All Authors**: No reported disclosures

